# Acute Ischemic Stroke in 2026: From Time to Penumbra—An Updated Narrative Review of Reperfusion Strategies with a Latin American Implementation Perspective

**DOI:** 10.3390/jcm15166496

**Published:** 2026-08-21

**Authors:** Danilo Alejandro Solarte Ordoñez, Jose Leonel Zambrano Urbano, Harold Enrique Vasquez Ucros, Ana Gabriela Cruz Suarez, Angie Estefanía Arcos Bastidas, Juan David Camacho Bolaños, Darío S. López Delgado, Angela Catalina Vallejo Cajigas, Oriana Rivera-Lozada, Cesar Bonilla Asalde, Joshuan J. Barboza

**Affiliations:** 1 Faculty of Medicine, Universidad Cooperativa de Colombia, Campus Pasto, Pasto 520001, Nariño, Colombia; daniloalejo7@gmail.com (D.A.S.O.); jose.zambrano@hotmail.com (J.L.Z.U.); haenvau@gmail.com (H.E.V.U.); darkeio92@gmail.com (D.S.L.D.); 2 Emergency Department, Fundación Hospital San Pedro, Pasto 520003, Nariño, Colombia; agabi08091011@gmail.com; 3 Semillero de Investigación AMEC, Universidad Cooperativa de Colombia, Campus Pasto, Pasto 520001, Nariño, Colombia; estefarcosbas@gmail.com (A.E.A.B.); camacho0703@hotmail.com (J.D.C.B.); 4 Grupo de Investigación en Ciencias de la Salud (GICIENSA), Universidad de Nariño, Pasto 520001, Nariño, Colombia; 5 Neurology Department, Hospital Universitario Departamental de Nariño, Pasto 520001, Nariño, Colombia; catalinavallejocajigas@gmail.com; 6 Vicerrectorado de Investigación, Universidad Señor de Sipán, Chiclayo 14000, Peru; riveraoriana@uss.edu.pe (O.R.-L.); bonasal@gmail.com (C.B.A.)

**Keywords:** ischemic stroke, thrombolysis, thrombectomy, posterior circulation, extended windows, tenecteplase

## Abstract

**Background/Objective:** The management of acute ischemic stroke (AIS) has evolved from rigid time-based treatment paradigms toward tissue-based selection guided by advanced neuroimaging, thereby expanding eligibility for reperfusion therapies. To provide an updated narrative review of acute ischemic stroke (AIS) classification and management, with emphasis on extended therapeutic windows for intravenous thrombolysis (IVT) and endovascular therapy (EVT), bridging strategies, posterior circulation stroke, and implementation challenges in Latin America and other resource-constrained settings. **Methods:** A structured narrative review was conducted using the PubMed/MEDLINE, Embase, Scopus, and LILACS databases, covering the period from 2013 to 2025. **Results:** Sixty-three studies were selected from 412 records and categorized into etiologic classification, extended-window thrombolysis, EVT and bridging therapy, and posterior circulation stroke. Current evidence supports imaging-guided IVT beyond 4.5 h and EVT up to 24 h in selected patients with salvageable brain tissue, including some individuals with large infarct cores. Recent trials also support EVT for basilar artery occlusion. Tenecteplase offers practical workflow advantages in many centers, particularly where transfer delays and limited access to advanced imaging constrain timely reperfusion decisions. **Conclusions:** Contemporary AIS management is increasingly guided by pathophysiology and imaging rather than strict time thresholds. However, improving outcomes in middle- and low-income settings requires the implementation of adapted clinical algorithms, strengthening of stroke care networks, and optimization of referral pathways.

## 1. Introduction

Ischemic stroke (IS) is the most common type of stroke, accounting for approximately 65–80% of cases worldwide [1]. It represents one of the most critical neurological emergencies, as the sudden occlusion of a cerebral artery leads to ischemia and neuronal death within minutes, resulting in substantial morbidity and mortality [2]. Over recent decades, the global burden of stroke has increased steadily and is projected to remain among the leading causes of death through 2030 [3]. Current estimates indicate that nearly 12 million new strokes occur annually and that one in four adults will experience a stroke during their lifetime. Overall, stroke (both ischemic and hemorrhagic) is the second leading cause of death globally, accounting for more than 7 million deaths each year, and the leading cause of disability in older adults, generating a significant health and economic burden across countries of all income levels [1,4].

In middle-income countries, particularly in Latin America, ischemic stroke has become a major public health concern. In Colombia, cerebrovascular diseases rank among the top five causes of mortality, with more than 24,000 deaths reported in 2021, over 80% of which were due to ischemic stroke. This burden reflects marked geographic and social disparities, including higher mortality rates in rural areas, poorer outcomes among women, and significant barriers to timely access to stroke units and reperfusion therapies [5,6].

Reperfusion therapy, including intravenous thrombolysis (IVT) and mechanical thrombectomy, has been the cornerstone of acute ischemic stroke treatment since its approval in 1996 [7]. Traditionally, therapeutic windows have been defined as up to 4.5 h for IVT and up to 6 h for mechanical thrombectomy, based on time and clinical–radiological criteria [8,9]. In addition, international standards emphasize key time-based performance metrics that directly influence prognosis, such as onset-to-door ≤ 60 min, door-to-needle ≤ 60 min, door-to-groin ≤ 90 min, and door-in-door-out ≤ 30 min for interhospital transfer [9,10,11]. However, the implementation of these strategies and time targets remains limited in many middle-income countries.

Despite strong evidence supporting extended therapeutic windows, a significant “implementation gap” persists in routine clinical practice, particularly in resource-limited regions such as Latin America. In real-world settings, the challenge is no longer only whether patients are eligible for treatment, but how healthcare systems can be organized to ensure continuous (24/7) access to advanced imaging and endovascular expertise. In this context, the concept of “time is brain” must be complemented by a systems-based perspective, “system is brain”, highlighting the importance of efficient healthcare organization in achieving optimal outcomes.

Data from the RES-Q registry (2017–2021), which included 7963 patients across 25 Colombian hospitals, demonstrated limited availability of reperfusion therapies: intravenous thrombolysis was performed in 14.5% of patients, mechanical thrombectomy in 3.0%, and combined therapy in only 2.4%. This data showed limited use of reperfusion therapies and substantial delays across the stroke pathway, with marked regional disparities, particularly in rural areas [5]. These findings are consistent with broader evidence showing that prehospital triage and system-level organization strongly influence access to timely reperfusion [12,13]. These findings underscore critical gaps in stroke care and highlight the need to expand therapeutic windows, implement emerging strategies to increase timely treatment, and optimize key performance metrics such as door-to-needle and door-to-puncture times. Accordingly, the aim of this review is to provide an updated and practical synthesis for specialized teams, promote evidence-based decision-making, and contribute to the standardization of ischemic stroke management.

## 2. Methods

This study was conducted as a narrative review. Although a structured search strategy was applied to identify relevant literature, this work does not meet the criteria for a systematic review, and the findings should be interpreted accordingly. The objective was to provide a comprehensive and clinically oriented synthesis of current evidence rather than formal evidence grading or meta-analytic assessment.

To enhance transparency, a structured literature search was performed in the PubMed/MEDLINE, Embase, Scopus, and LILACS databases, covering the period from January 2013 to 30 June 2025. The search strategy included combinations of Medical Subject Headings (MeSH) and DeCS terms such as “ischemic stroke,” “extended window,” “thrombectomy,” “intravenous thrombolysis,” “endovascular therapy,” and “posterior circulation stroke,” along with their Spanish equivalents, using Boolean operators (AND, OR). The search was complemented by a manual review of reference lists from key articles.

Study selection was based on relevance to the topic and clinical applicability. Included sources comprised randomized controlled trials, meta-analyses, systematic reviews, key observational studies, and major international and national clinical practice guidelines addressing acute ischemic stroke in adult populations (Included sources comprised randomized controlled trials, meta-analyses, systematic reviews, key observational studies, and major international and national clinical practice guidelines addressing acute ischemic stroke in adult populations). No formal screening protocol (e.g., PRISMA flow diagram) or duplicate independent review process was applied.

No formal risk-of-bias assessment or quantitative synthesis was performed. Instead, the evidence was interpreted qualitatively, with emphasis on consistency across studies, clinical relevance, and applicability to real-world settings, particularly in Latin America. Given the narrative nature of this review, conclusions should be understood as an expert-informed synthesis rather than graded recommendations.

The SANRA (Scale for the Assessment of Narrative Review Articles) framework was used as a general guide to improve clarity, structure, and methodological transparency during manuscript development; however, no formal scoring or itemized reporting was conducted.

Based on this synthesis, a pragmatic diagnostic–therapeutic algorithm was developed to support decision-making in acute ischemic stroke management within emergency departments and stroke units in Latin American contexts. This algorithm is intended as a contextualized clinical tool rather than a prescriptive guideline.

## 3. Results

Following the initial search, 423 records were identified. After duplicate removal and application of predefined inclusion criteria, 74 studies were selected to support this review. The selected studies were organized into four main thematic areas:

- Prehospital stroke recognition and early clinical identification, including validated screening tools and triage strategies for timely diagnosis and referral, particularly relevant in resource-constrained environments.
- Current classification and etiological evaluation of ischemic stroke, including established entities such as ESUS (Embolic Stroke of Undetermined Source) and recent advances in etiological stratification.
- Thrombolysis in extended time windows, supported by recent clinical trials and its applicability in settings using advanced imaging-based selection.
- Endovascular therapy, including extended therapeutic windows and the evolving role of combined strategies (bridging therapy vs. direct EVT), with a focus on comparative efficacy and patient selection.
- Posterior circulation stroke, emphasizing contemporary reperfusion evidence and the pathophysiological and diagnostic particularities of this vascular territory.

### 3.1. Prehospital Stroke Recognition and Early Clinical Identification

Early recognition of acute ischemic stroke in the prehospital setting is a critical determinant of timely reperfusion and improved clinical outcomes. Delays in stroke identification are directly associated with worse functional outcomes, higher rates of disability, and increased mortality, as they prolong time to reperfusion and reduce the likelihood of salvaging viable brain tissue [14]. While advances in imaging and endovascular therapies have expanded treatment windows, delays in initial stroke recognition remain a major barrier, particularly in low- and middle-income regions such as Latin America [15,16].

Prehospital stroke recognition relies on simple, validated clinical tools that can be applied by emergency medical services (EMS) personnel and non-specialized healthcare providers. The most widely used screening tools include the FAST (Face, Arm, Speech, Time) scale and its expanded versions, such as BE-FAST (Balance, Eyes, Face, Arm, Speech, Time), which improve sensitivity for posterior circulation stroke by incorporating balance and visual symptoms [17]. These tools are designed for rapid identification of suspected stroke and immediate activation of stroke pathways [18].

In addition to general stroke recognition, several prehospital scales have been developed to identify patients with a high probability of large vessel occlusion (LVO), who may benefit from direct transfer to comprehensive stroke centers capable of performing endovascular therapy. Commonly used LVO screening tools include the Los Angeles Motor Scale (LAMS), Rapid Arterial Occlusion Evaluation (RACE) scale, Cincinnati Prehospital Stroke Severity Scale (CPSSS), and FAST-ED score. These scales assess varying combinations of motor deficit, cortical signs, and level of consciousness, with moderate-to-high accuracy for predicting LVO in the field [19,20,21].

In resource-constrained settings, the implementation of these tools must be adapted to local capabilities. Simpler scales such as FAST or BE-FAST may be more feasible for widespread use, particularly in rural or underserved areas with limited EMS training. In contrast, more detailed LVO scales may be incorporated in urban systems with structured prehospital care and access to referral networks such as the LAMS [19].

From a systems perspective, early stroke identification should be integrated with prehospital triage strategies, including decisions regarding transport destination (primary vs. comprehensive stroke center) and activation of stroke teams prior to hospital arrival [22,23,24]. These strategies are essential to reduce delays in door-to-needle and door-to-groin times and to optimize the use of available resources.

**Figure 1:**
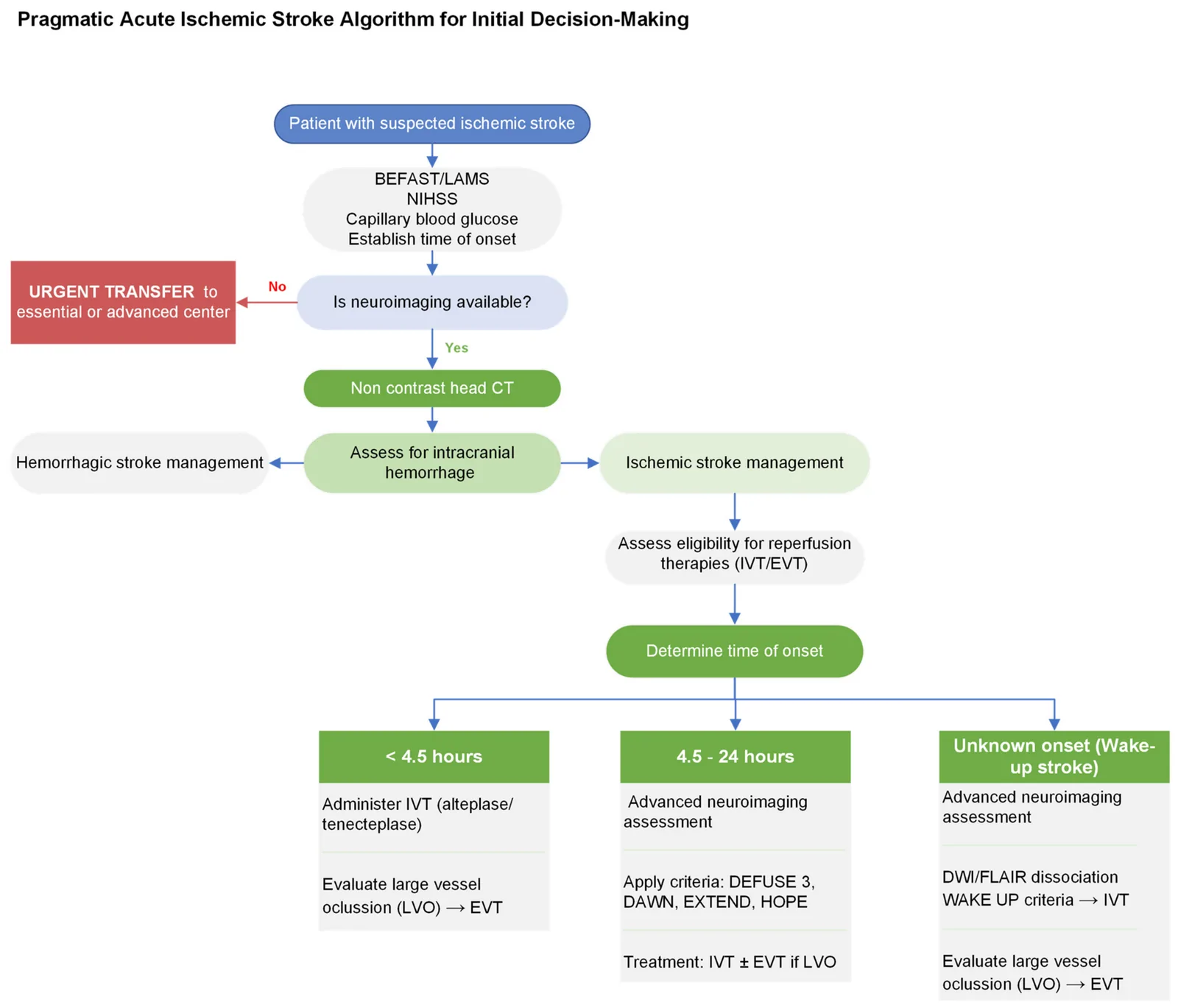
Pragmatic approach to prehospital assessment, triage, and initial imaging-based treatment pathways for acute ischemic stroke based on resource availability in Latin America. This figure presents a resource-adapted, tiered approach to acute ischemic stroke management, stratifying care according to the availability of neuroimaging and endovascular therapy, and emphasizing early treatment and structured transfer pathways. Abbreviations: IVT: Intravenous thrombolysis, EVT: Endovascular thrombectomy, LVO: Large vessel occlusion. Clinical Considerations: 1. LVO suspicion: NIHSS ≥ 6–10, 2. Prioritize IVT when EVT is not immediately available, 3. Early transfer is critical at all levels.

In the context of Latin America, strengthening prehospital stroke recognition represents a key opportunity to improve access to reperfusion therapies. Training EMS personnel, standardizing the use of validated scales, and integrating prehospital assessment into regional stroke networks are fundamental steps toward reducing treatment delays and improving outcomes [25]. A conceptual framework for early recognition and initial decision-making is illustrated in Figure 1, which outlines a pragmatic approach to prehospital assessment, triage, and initial imaging-based treatment pathways adapted to variable resource settings in Latin America.

### 3.2. Current Classification and Etiological Evaluation of Ischemic Stroke

The etiological classification of ischemic stroke has traditionally been based on the TOAST criteria (Trial of ORG 10172 in Acute Stroke Treatment), which identify five main subtypes [26]:

- Large-artery atherosclerosis (LAA): Associated with ≥50% stenosis of the carotid or major cerebral arteries; accounts for approximately 15–20% of cases.
- Cardioembolic (CE): Resulting from embolism originating in the heart (e.g., atrial fibrillation, intracavitary thrombi, prosthetic valves, endocarditis), typically characterized by sudden onset, larger infarct volumes, and poorer prognosis; represents 20–30% of cases.
- Small-vessel occlusion (lacunar): Caused by lipohyalinosis or microatheromatosis affecting penetrating arteries, leading to infarcts <15 mm in regions such as the basal ganglia or pons; often associated with lacunar syndromes; accounts for 20–25% of cases.
- Other determined etiologies: Includes less common causes such as arterial dissections, vasculitis, prothrombotic states, genetic conditions, or infections; represents <5% of cases.
- Undetermined etiology (cryptogenic or multiple): Defined when no cause is identified despite comprehensive evaluation, or when multiple potential mechanisms coexist without a clear predominance; reported in up to 30–35% of cases.

This classification remains clinically relevant, as it guides secondary prevention strategies (e.g., anticoagulation, antiplatelet therapy, and risk factor control) and provides prognostic information. For instance, cardioembolic strokes are typically more extensive and associated with poorer outcomes, whereas lacunar strokes generally follow a more favorable clinical course [27].

Despite its clinical utility, the TOAST system has important limitations, particularly in complex cases. Up to 10–40% of strokes remain without an identified cause despite extensive diagnostic evaluation. Moreover, its unidimensional structure requires the assignment of a single predominant etiology, even when multiple mechanisms may coexist, potentially leading to diagnostic oversimplification or misclassification [26].

Recent studies have demonstrated limited diagnostic concordance between etiologies assigned by treating physicians and those determined by expert panels with access to comprehensive investigations, with agreement rates of approximately 61%. Common sources of misclassification include overestimation of cardioembolic origin based solely on subclinical atrial fibrillation and misattribution to atherosclerosis in cases of moderate carotid stenosis without evidence of plaque instability [28].

To address these limitations, more flexible classification systems such as the ASCOD model (Atherosclerosis, Small vessel disease, Cardiac pathology, Other causes, Dissection) have been proposed. This multidimensional framework assigns a graded level of causality (1–3) to each potential mechanism, providing a more nuanced representation of stroke etiology and supporting more individualized approaches to secondary prevention [29].

Another limitation of the TOAST classification is its limited ability to adequately capture specific subgroups within cryptogenic stroke, particularly ESUS (Embolic Stroke of Undetermined Source). ESUS is an established clinical construct that includes non-lacunar infarcts with embolic imaging features (e.g., cortical, multiple, or bilateral lesions) in the absence of major cardioembolic sources, significant atherosclerosis, or small-vessel disease [30].

Emerging evidence suggests that a substantial proportion of ESUS cases may be attributable to previously undetected paroxysmal atrial fibrillation (AF). In this context, the EMBRACE and CRYSTAL-AF trials demonstrated that prolonged cardiac monitoring significantly increases AF detection. In EMBRACE, 30-day external monitoring identified AF episodes ≥30 s in 16.1% of patients compared with 3.2% using conventional 24 h Holter monitoring (RR 5.08; 95% CI 1.51–17.11; *p* < 0.002), leading to higher rates of anticoagulation (18.6% vs. 11.1%; absolute difference 7.5%; *p* = 0.01) [31]. Similarly, CRYSTAL-AF reported cumulative AF detection rates of 8.9% at 6 months, 12.4% at 12 months, and 30.0% at 36 months with implantable monitors, compared with 1.4% in control groups, with numbers needed to screen (NNS) of 14, 10, and 4, respectively [32].

However, the detection of subclinical atrial fibrillation (AF) does not necessarily justify immediate empirical anticoagulation. The NAVIGATE-ESUS trial showed no reduction in recurrent stroke with rivaroxaban and an increased risk of major bleeding, while RE-SPECT ESUS demonstrated no significant difference between dabigatran and aspirin [33,34]. These findings highlight the importance of confirming AF before initiating anticoagulant therapy.

Beyond cardiac monitoring, additional diagnostic tools have expanded the evaluation of cryptogenic stroke. Carotid plaque MRI can identify non-stenotic yet vulnerable plaques, while MR angiography, transesophageal echocardiography with bubble study, and cardiac MRI may detect subclinical embolic sources or paradoxical embolism [26].

From a Latin American perspective, registries such as CARMEN and RECCLA have reported higher proportions of cardioembolic strokes associated with underdiagnosed AF, as well as atherosclerotic strokes linked to inadequate risk factor control [35]. Colombian data from the RES-Q registry (2025) and the national stroke protocol are consistent with these findings [5,12].

To mention, each classification system has distinct clinical value: TOAST remains the global standard for routine practice, ASCOD provides a multidimensional framework for greater etiological precision, and ESUS represents an established construct within cryptogenic stroke that supports research and more individualized prevention strategies. The integrated use of these approaches enables more accurate etiological characterization and more tailored therapeutic decision-making.

### 3.3. Thrombolysis in the Era of Extended Windows

Intravenous thrombolysis (IVT) with tissue plasminogen activator (rtPA, most commonly alteplase) has been the standard treatment for acute ischemic stroke in the early time window for decades. Since the landmark NINDS trial (1995), a clear functional benefit has been demonstrated when alteplase is administered within the first 3 h, increasing the probability of recovery without disability at three months by approximately 30% (OR 1.7), with a higher risk of symptomatic intracerebral hemorrhage (6.4% vs. 0.6%) [7]. Subsequently, the ECASS III trial (2008) extended the therapeutic window to 4.5 h, showing improved functional outcomes (mRS 0–1 in 52.4% vs. 45.2% with placebo; OR 1.34; *p* = 0.04), with a modest increase in symptomatic hemorrhage (2.4% vs. 0.2%) but no increase in mortality [8].

However, a substantial proportion of patients present beyond this time window or with unknown symptom onset (e.g., wake-up stroke). Over the past decade, this limitation has driven a paradigm shift in patient selection: treatment decisions are no longer based solely on chronological time but increasingly incorporate imaging-based approaches. Importantly, these strategies include both time-surrogate imaging methods and physiological assessment of tissue viability, using advanced imaging to identify patients who may benefit from reperfusion beyond 4.5 h [13]. Several key studies have investigated the extension of thrombolysis into later time windows in carefully selected patients, including:

- WAKE-UP (NEJM 2018): This trial included 503 patients with stroke of unknown onset (typically upon awakening), selected using MRI demonstrating a diffusion-FLAIR mismatch (i.e., a lesion visible on DWI but not on FLAIR), which serves as a surrogate marker of early time window (<4.5–6 h) rather than direct assessment of penumbral tissue. Alteplase administered within 4.5 h from symptom recognition significantly improved functional independence (mRS 0–1: 53.3% vs. 41.8%; OR 1.62; *p* = 0.003), with an absolute difference of approximately 11–12%. Although symptomatic intracranial hemorrhage was more frequent in the treatment group, the difference was not statistically significant (2.0% vs. 0.4%), and no increase in mortality was observed (4.1% vs. 1.2%; *p* = 0.07). These findings support the use of imaging-based selection in patients with unknown onset, but within a time-surrogate framework rather than true penumbra-based selection [36].
- EXTEND (NEJM 2019): This trial evaluated 225 patients treated between 4.5 and 9 h after symptom onset (or with wake-up stroke), selected based on perfusion mismatch criteria (core <70 mL, penumbra >10 mL, mismatch ratio ≥1.2) using CT perfusion (CTP) or MRI (PWI/DWI). Alteplase significantly increased functional independence (mRS 0–2: 35.4% vs. 29.5%; adjusted RR 1.44; *p* = 0.04), although it was associated with a higher rate of symptomatic intracranial hemorrhage (6.2% vs. 0.9%), without a significant increase in mortality [37]. These results demonstrated the feasibility of extending thrombolysis up to 9 h using imaging-based selection, although the magnitude of benefit was more modest compared with the standard time window (NNT = 17) [38].
- HOPE (JAMA 2025): This recent trial evaluated the safety and efficacy of alteplase administered up to 24 h after symptom onset in imaging-selected patients. A total of 372 patients were included, meeting viability criteria similar to those used in EXTEND (core <70 mL, penumbra/core ratio ≥1.2, and mismatch volume >10 mL), and excluding candidates for thrombectomy. Alteplase was associated with higher rates of functional independence (mRS 0–1 at 90 days: 49.5% vs. 35.5%; absolute difference 13.9%; *p* = 0.004), with a symptomatic intracranial hemorrhage rate of 3.8% and no increase in mortality. Although most treated patients were within the first 9 h, the trial supports the safety and efficacy of imaging-guided thrombolysis within an extended window of up to 24 h [39].
- Meta-analysis (Stroke 2025): These findings were further supported by a meta-analysis published in Stroke (2025), which included eight randomized trials (excluding HOPE) and 1742 patients treated with thrombolysis beyond 4.5 h (up to 24 h), excluding those undergoing thrombectomy. The pooled results confirmed the clinical benefit of an imaging-based selection strategy: thrombolysis increased rates of excellent recovery (mRS 0–1: 44% vs. 36%; OR 1.43; *p* = 0.0005) and improved functional outcomes (mRS 0–2; OR 1.36; *p* = 0.002). Greater benefit was observed with perfusion-based selection (OR 1.45; 95% CI 1.08–1.94) compared with DWI–FLAIR mismatch selection (OR 1.34; 95% CI 0.94–1.91). Additionally, the effect appeared more pronounced with tenecteplase (OR 1.47; 95% CI 1.06–2.04) than with alteplase (OR 1.38; 95% CI 1.08–1.78). Symptomatic intracranial hemorrhage rates were below 5–6%, and no significant differences in mortality were observed, supporting the concept that imaging-defined tissue viability can supersede strict time-based treatment thresholds [40].

Contemporary evidence from WAKE-UP, EXTEND, HOPE, and related studies (Table 1) has driven a paradigm shift in acute ischemic stroke management. Patients previously excluded based solely on time criteria may benefit from intravenous thrombolysis (IVT) when advanced imaging demonstrates the presence of salvageable tissue (ischemic penumbra) [12]. Accordingly, the traditional concept of “time is brain” has evolved toward a more pathophysiological framework, emphasizing that “tissue, and particularly the penumbra, is brain” [12]. Table 1 summarizes major clinical trials assessing thrombolysis in standard and extended windows, highlighting advanced imaging selection criteria and relevant outcomes.

Both the AHA/ASA 2026 and ESO 2023 guidelines have incorporated these recommendations. The AHA/ASA guidelines suggest considering intravenous thrombolysis in patients with unknown time of onset who demonstrate a DWI–FLAIR mismatch on MRI, as well as in those presenting up to 9 h after symptom onset with favorable perfusion profiles on CTP or MRI (PWI/DWI) (Class IIb) [10]. Similarly, the ESO 2023 guidelines support thrombolysis in extended time windows (grade B, moderate-quality evidence) in patients selected using imaging-based physiological criteria, including perfusion–core mismatch (EXTEND criteria) or DWI–FLAIR mismatch (WAKE-UP criteria), particularly in centers with advanced imaging capabilities and individualized clinical assessment [11].

Furthermore, recent evidence from the HOPE trial (JAMA 2025) and the meta-analysis by Günkan et al. (Stroke 2025) suggests that imaging-guided thrombolysis may be both effective and safe up to 24 h after symptom onset, thereby substantially expanding the therapeutic window for pharmacological reperfusion [39,40]. Although these findings require cautious interpretation and further validation across diverse populations, they provide a rationale for considering thrombolysis within a 24 h window in carefully selected patients. This perspective is partially reflected in recent guidelines, which suggest that IV thrombolysis may be considered in highly selected cases, particularly in patients with large-vessel occlusion, imaging-defined salvageable tissue, and no access to endovascular therapy, although this remains a conditional recommendation [10]. Broader adoption of this approach will depend on further evidence and formal incorporation into future guideline updates.

**Figure 2:**
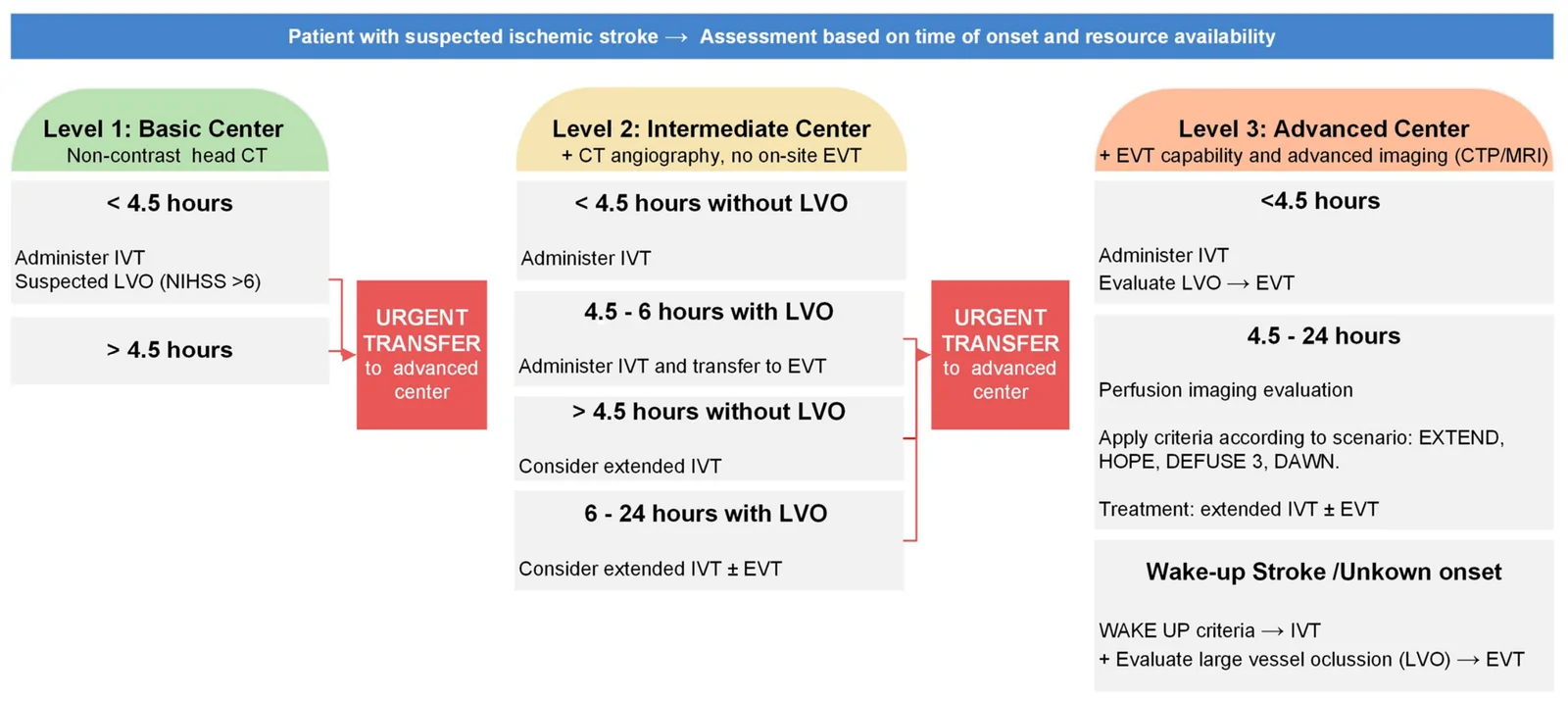
Tiered decision-making framework for acute ischemic stroke based on resource availability in Latin America. This figure illustrates a simplified, time-based approach for the initial evaluation and management of acute ischemic stroke, integrating eligibility for intravenous thrombolysis (IVT) and endovascular therapy (EVT) according to time from symptom onset and imaging findings. Abbreviations and Imaging Criteria CT: computed tomography, BEFAST: acronym. for recognizing a stroke (B—Balance: Watch for sudden loss of balance. E—Eyes: Check for vision loss. F—Face: Look for an uneven smile. A—Arm: Check if one arm is weak), NIHSS: National Institute of Health Stroke Score, LVO: Large vessel occlusion (ICA, MCA M1, ACA A1, Basilar artery). Extended Window Thrombolysis Criteria (EXTEND): An extended window of 4.5 to 9 h: 1. Core < 70 mL, 2. Penumbra ≥ 10 mL, 3. Mismatch ratio ≥ 1.2. Late Window Thrombolysis Criteria (HOPE) An extended window of 4.5 to 24 h: 1. Core < 70 mL, 2. Penumbra ≥ 10 mL, 3. Mismatch ratio ≥ 1.2. For stroke onset 6–24 h before expected arterial access, and intracranial anterior circulation occlusion with a small-to-moderate ischemic use DAWN or DEFUSE 3 criteria to support EVT eligibility, DAWN (clinical–core mismatch): 1. Age < 80 years: NIHSS ≥ 10, core < 31 mL, 2. Age ≥ 80 years: NIHSS ≥ 10, core < 21 mL. DEFUSE 3: 1. Core < 70 mL, 2. Penumbra ≥ 15 mL, 3. Mismatch ratio ≥1.8. WAKE-UP: 1. DWI-positive lesion without FLAIR hyperintensity, 2. Estimated onset <4.5–6 h, 3. No extensive infarction.

Figure 2 illustrates a tiered decision-making framework for acute ischemic stroke based on resource availability in Latin America.

Importantly, in extended-window scenarios, intravenous thrombolysis (IVT) is generally considered when endovascular therapy (EVT) is not feasible (e.g., in non-large-vessel occlusions or in centers without immediate access to thrombectomy) [11]. In patients with large-vessel occlusion (LVO) who are eligible for late-window thrombectomy, EVT remains the preferred strategy. However, in early-window presentations (≤4.5 h), some protocols continue to recommend “bridging” thrombolysis prior to EVT when no contraindications are present [11]. In clinical practice, treatment decisions should be individualized, balancing the potential benefits of IVT against the risks, particularly when rapid mechanical reperfusion is already achievable [41]. These considerations, along with the evolving role of combined therapy, are discussed in the following section.

#### Tenecteplase and Single-Bolus Administration

Tenecteplase, a genetically modified variant of rtPA with greater fibrin specificity and a longer half-life, allows single-bolus administration, thereby substantially simplifying hospital workflows, particularly in emergency departments and during interhospital transfers in resource-limited settings. This advantage is especially relevant in environments where CT angiography is routinely used to assess collateral circulation [42]. Multiple studies have evaluated its efficacy and safety:

- EXTEND-IA TNK (NEJM 2018): This trial included patients with large-vessel occlusions (LVO) eligible for thrombectomy and demonstrated that tenecteplase at a dose of 0.25 mg/kg achieved higher rates of pre-thrombectomy reperfusion compared with alteplase (22% vs. 10%; OR 2.6; 95% CI 1.1–5.9). Tenecteplase was also associated with improved functional outcomes at 90 days, reflected by a more favorable mRS distribution (OR 1.7; 95% CI 1.0–2.8). Rates of symptomatic intracranial hemorrhage (sICH) were low and similar in both groups (1%) [43].
- EXTEND-IA TNK II (JAMA 2020): This trial compared tenecteplase at doses of 0.25 mg/kg versus 0.4 mg/kg and found no significant differences in reperfusion or functional outcomes (mRS at 90 days: RR 1.03; 95% CI 0.66–1.61), supporting 0.25 mg/kg as the optimal dose [44]. In larger trials, including TRACE-2 (Lancet 2023) and ATTEST-2 (Lancet 2024), tenecteplase at 0.25 mg/kg demonstrated non-inferiority to alteplase in terms of functional outcomes, with comparable rates of symptomatic intracranial hemorrhage (3–4%) and no differences in mortality at 90 days [45,46].

In extended windows, two recent NEJM 2024 trials provided key evidence for tenecteplase beyond 4.5 h:

- TIMELESS (NEJM 2024): This trial included 458 patients with large-vessel occlusion (LVO) and imaging evidence of salvageable tissue treated between 4.5 and 24 h after symptom onset. Tenecteplase demonstrated a favorable safety profile (sICH: 3.2% vs. 2.3%) and achieved higher rates of recanalization compared with standard care (76.7% vs. 63.9%). A trend toward improved functional outcomes was observed (mRS 0–2: 45.9% vs. 31.4%), although this difference did not reach statistical significance [47].
- TRACE-III (NEJM 2024): this trial showed that patients without access to endovascular thrombectomy, tenecteplase administered between 4.5 and 24 h after symptom onset was associated with reduced disability (mRS 0–1: 33.0% vs. 24.2%; RR 1.37; 95% CI 1.04–1.81; *p* = 0.03), with an acceptable safety profile (sICH: 3.0% vs. 0.8%) [48].

Overall, these trials support the non-inferiority of tenecteplase compared with alteplase, highlight its potential logistical advantages, and suggest benefit in extended, imaging-guided treatment windows for patients with large-vessel occlusion (LVO). This is particularly relevant in both EVT-capable centers and settings without immediate access to thrombectomy, common scenarios in Latin America, where “drip-and-ship” strategies may help reduce delays by streamlining in-hospital processes [43]. Accordingly, the ESO (2023) guidelines recommend tenecteplase at a dose of 0.25 mg/kg over alteplase 0.9 mg/kg in patients with large-vessel occlusion (LVO) presenting within 4.5 h who are eligible for intravenous thrombolysis (IVT) and are planned for endovascular thrombectomy (strong recommendation, moderate-quality evidence), primarily due to higher rates of early reperfusion and logistical advantages related to single-bolus administration [11,46]. However, These findings and current guideline recommendations suggest that in patients presenting within <4.5 h without LVO, both ESO (2023) and AHA/ASA 2026 indicate that tenecteplase 0.25 mg/kg may be considered as an alternative to alteplase; however, this remains a weaker recommendation (ESO: weak recommendation; AHA/ASA: Class IIb), reflecting comparable efficacy and safety alongside the practical advantage of simplified administration [10,11]. Overall, current evidence supports tenecteplase as a viable and operationally advantageous alternative to alteplase, particularly in LVO patients undergoing planned thrombectomy, while its broader adoption in other clinical scenarios continues to evolve.

### 3.4. Endovascular Therapy in Ischemic Stroke: Extended Windows and Bridging Therapy

Mechanical thrombectomy (EVT), performed using endovascular devices such as stent retrievers or direct aspiration systems, represents one of the most significant therapeutic advances since the introduction of rtPA [49]. It is primarily indicated for large-vessel intracranial occlusions, including the terminal internal carotid artery and proximal segments of the middle cerebral artery (MCA), where it achieves higher reperfusion rates compared with systemic thrombolysis alone [50].

In 2015, five landmark randomized controlled trials (MR CLEAN, ESCAPE, EXTEND-IA, SWIFT PRIME, and REVASCAT) consistently demonstrated that thrombectomy performed within the first 6 h, combined with IV thrombolysis in most cases, significantly improves functional independence compared with medical therapy alone [49]. A pooled meta-analysis (*n* = 1287) estimated a number needed to treat (NNT) of three to seven to achieve one additional favorable functional outcome (mRS 0–2), establishing EVT as the standard of care for LVO ischemic stroke in the early time window [9]. Accordingly, current guidelines recommend urgent thrombectomy within 6 h of symptom onset, ideally preceded by IV thrombolysis in eligible patients [10,11].

#### 3.4.1. Extended Windows for Endovascular Therapy (6–24 h)

Following the success of early-window thrombectomy, the next critical question was whether patients presenting later could still benefit from intervention if salvageable brain tissue remained [50]. Two pivotal trials published in 2018 fundamentally changed clinical practice:

- DAWN (NEJM 2018): This trial included 206 patients with occlusion of the terminal internal carotid artery or proximal middle cerebral artery (MCA) treated between 6 and 24 h from last known well. Patients were selected based on a clinical–imaging mismatch (severe neurological deficit with a small infarct core). Eligibility criteria included: age ≥80 years with NIHSS ≥10 and core volume <21 mL; or age <80 years with NIHSS ≥10 and core <31 mL (or NIHSS ≥20 and core <51 mL). The trial was stopped early due to efficacy, demonstrating functional independence (mRS 0–2) in approximately 50% of patients undergoing thrombectomy versus 13–19% with medical therapy alone (OR 4.5), without significant increases in mortality or symptomatic intracranial hemorrhage, supporting benefit up to 24 h in highly selected patients [51].
- DEFUSE 3 (NEJM 2018): This trial included 182 patients treated between 6 and 16 h after symptom onset with ICA or MCA M1 occlusion, selected using perfusion imaging (CT or MRI with RAPID software: core <70 mL, penumbra ≥15 mL, mismatch ratio ≥1.8). Similarly to DAWN, the trial was stopped early after demonstrating benefit. Thrombectomy significantly improved functional outcomes, with independence (mRS 0–2) achieved in 45% of patients compared with 17% in the medical therapy group (*p* < 0.001; NNT= 3–4) [52].

However, the translation of these findings into routine clinical practice requires careful consideration. Both trials relied on advanced imaging (CT perfusion or MRI) and highly controlled selection criteria, which may not be consistently available in many real-world settings, particularly in low- and middle-income countries. In addition, interhospital transfer delays, common in “drip-and-ship” models, may reduce or negate the potential benefit of late-window thrombectomy, emphasizing that extended time windows should not be interpreted as justification for delayed care.

Both trials confirmed that advanced imaging selection, clinical–core mismatch or perfusion–core mismatch can identify patients with viable tissue beyond traditional windows. These results consolidated the principle that physiology can outweigh time and established the basis for current recommendations extending EVT up to 24 h.

Accordingly, current guidelines reflect this balance between evidence and feasibility. The AHA/ASA guidelines recommend thrombectomy up to 16 h (Class I) and consider it reasonable up to 24 h (Class IIa) in patients meeting DAWN or DEFUSE 3 eligibility criteria [10]. Similarly, ESO (2023) supports late-window EVT in carefully selected patients based on advanced imaging, while emphasizing the importance of appropriate infrastructure and expertise [11]. In this context, extended-window thrombectomy should be understood not as a universal strategy, but as a targeted intervention for selected patients within organized stroke systems capable of timely imaging, decision-making, and transfer.

Patient selection remains critical, as late reperfusion may not benefit all patients and, in some cases, may be associated with harm [50]. Nevertheless, emerging evidence has expanded the range of patients who may be eligible for late-window EVT under specific clinical and imaging-based considerations:

- Occlusion location: The strongest evidence supports EVT in anterior circulation large-vessel occlusions (LVO), particularly involving the internal carotid artery (ICA) or proximal middle cerebral artery (MCA). For basilar artery occlusion (posterior circulation), the evidence base has evolved more recently and is addressed in a dedicated section below. Earlier guidelines suggested that EVT may be considered in this context due to the high mortality associated with untreated basilar artery occlusion, although this recommendation was based on lower levels of evidence (conditional recommendation, Class IIa/B) [11,50].
- Large ischemic cores: Recent trials have expanded EVT eligibility to include patients with large infarct cores (e.g., >70 mL or ASPECTS 3–5), a group previously considered to have a poor prognosis.
  *(I)*  *SELECT2 (NEJM 2023):* included 352 patients with anterior circulation LVO and large infarcts defined by ASPECTS 3–5 on non-contrast CT or infarct volume ≥50 mL on CTP or MRI (low ADC). EVT significantly reduced disability, with mRS 0–3 achieved in 46% of patients compared with 30% in the medical therapy group (OR 1.97; 95% CI 1.21–3.20; *p* = 0.003), without a significant increase in symptomatic intracranial hemorrhage or mortality, although treatment effects were attenuated with increasing infarct volume [53].
  *(II)*  *ANGEL-ASPECT (NEJM 2023):* an Asian multicenter trial including 456 patients, defined large infarct cores using ASPECTS 3–5 on CT or volumes of 70–100 mL on CTP/MRI, without requiring formal penumbral mismatch. EVT improved the overall functional outcome distribution at 90 days (OR 1.37; 95% CI 1.11–1.69; *p* = 0.004), with a number needed to treat (NNT) of 8 to reduce one level of disability [54]. Subsequent analyses suggest that better outcomes may be associated with concordant clinical–imaging profiles (e.g., high NIHSS with large core) and favorable perfusion parameters, such as a hypoperfusion-to-core ratio ≥1.8 and penumbra volume ≥15 mL [55]. However, these findings should be interpreted cautiously, as treatment effects are smaller than in patients with small infarct cores and may depend on careful patient selection.
- Other selection considerations: Current guidelines recommend considering baseline functional status, typically defined as a pre-stroke mRS of 0–1, and a minimum NIHSS score of ≥6. There is no strict upper limit for NIHSS; patients with very severe deficits (NIHSS >20) may still benefit from early intervention, although some centers place greater emphasis on imaging to exclude extensive established infarction [50]. Age alone should not be considered an exclusion criterion, as benefit has been demonstrated in patients older than 80 years, albeit with lower rates of functional independence [50]. Regarding imaging criteria, an ASPECTS ≥6 is generally required in early time windows. In extended-window scenarios, however, advanced imaging with CT perfusion or MRI is often necessary to quantify infarct core and penumbral tissue, thereby guiding patient selection more precisely [10,11,50].

These findings suggest that EVT may provide benefit even in patients with extensive infarct cores, although rates of full functional independence remain lower compared with those observed in patients with smaller core volumes [50]. Importantly, no significant increase in symptomatic intracranial hemorrhage or mortality has been observed, supporting the relative safety of this approach when guided by careful quantitative imaging selection [56]. Nevertheless, the magnitude of benefit appears more modest, reinforcing the importance of individualized patient selection.

Overall, current evidence supports mechanical thrombectomy for LVO ischemic stroke within 6 h, and up to 24 h in carefully selected patients meeting DAWN or DEFUSE 3–type imaging profiles with evidence of salvageable tissue [57]. When feasible, EVT should be performed in addition to intravenous thrombolysis (IVT) in eligible patients within ≤4.5 h, unless specific contraindications or clinical considerations justify omission of IVT [11].

#### 3.4.2. Endovascular Therapy Alone vs. Combined with IV Thrombolysis (Bridging Therapy)

A key clinical dilemma is whether patients with large-vessel occlusion (LVO) presenting within the intravenous thrombolysis (IVT) window (<4.5 h) should receive thrombolysis prior to thrombectomy (bridging therapy) or proceed directly to the angiography suite for endovascular treatment (direct EVT) [41].

Recent randomized trials have explored this question, with heterogeneous results across different populations and study designs. The DIRECT-MT trial (NEJM 2020), one of the first to address this issue, demonstrated non-inferiority of direct EVT for 90-day functional outcomes; however, the margin of non-inferiority was narrow, and bridging therapy was associated with higher rates of early recanalization, without significant differences in mortality but with a slightly increased risk of symptomatic intracranial hemorrhage [58]. Subsequent Asian trials, including DEVT, reported similar findings supporting non-inferiority of direct EVT [59]. Whereas the smaller SKIP trial (using low-dose alteplase) did not formally demonstrate non-inferiority, likely due to limited statistical power despite comparable outcomes [60]. In contrast, European trials such as MR CLEAN-NO IV and SWIFT-DIRECT did not confirm non-inferiority of direct EVT and showed a non-significant trend favoring bridging therapy for functional outcomes [61,62]. A meta-analysis of 13 studies (*n* = 3985) reported similar rates of functional independence and mortality between both strategies; however, bridging therapy was associated with higher successful recanalization rates and a tendency toward improved overall outcomes, while direct EVT was associated with slightly lower rates of symptomatic intracranial hemorrhage in some analyses [63].

Overall, the available evidence suggests clinical equipoise between strategies, with important trade-offs between early reperfusion benefits and hemorrhagic risk. Current data indicate that direct EVT is not inferior, but also not clearly superior, to combined therapy. These findings should be interpreted in the context of differences in study populations, healthcare systems, and workflow times, which may influence their applicability to real-world practice, particularly in resource-constrained settings.

Importantly, intravenous thrombolysis may facilitate partial reperfusion of distal branches or even complete recanalization prior to EVT. Accordingly, both AHA/ASA and ESO guidelines recommend that thrombolysis should not be omitted in eligible patients when its administration does not delay endovascular treatment [10,11].

In clinical practice, an individualized approach is generally adopted. When intravenous thrombolysis (IVT) can be administered without delaying thrombectomy, it is typically given prior to EVT. Conversely, when significant delays to endovascular treatment are anticipated, such as during interhospital transfer, bridging thrombolysis with alteplase (or tenecteplase) may be particularly important [11]. Direct EVT is usually reserved for selected situations, including contraindications to thrombolysis or presentation beyond the IVT window but within the thrombectomy window [11].

**Figure 3:**
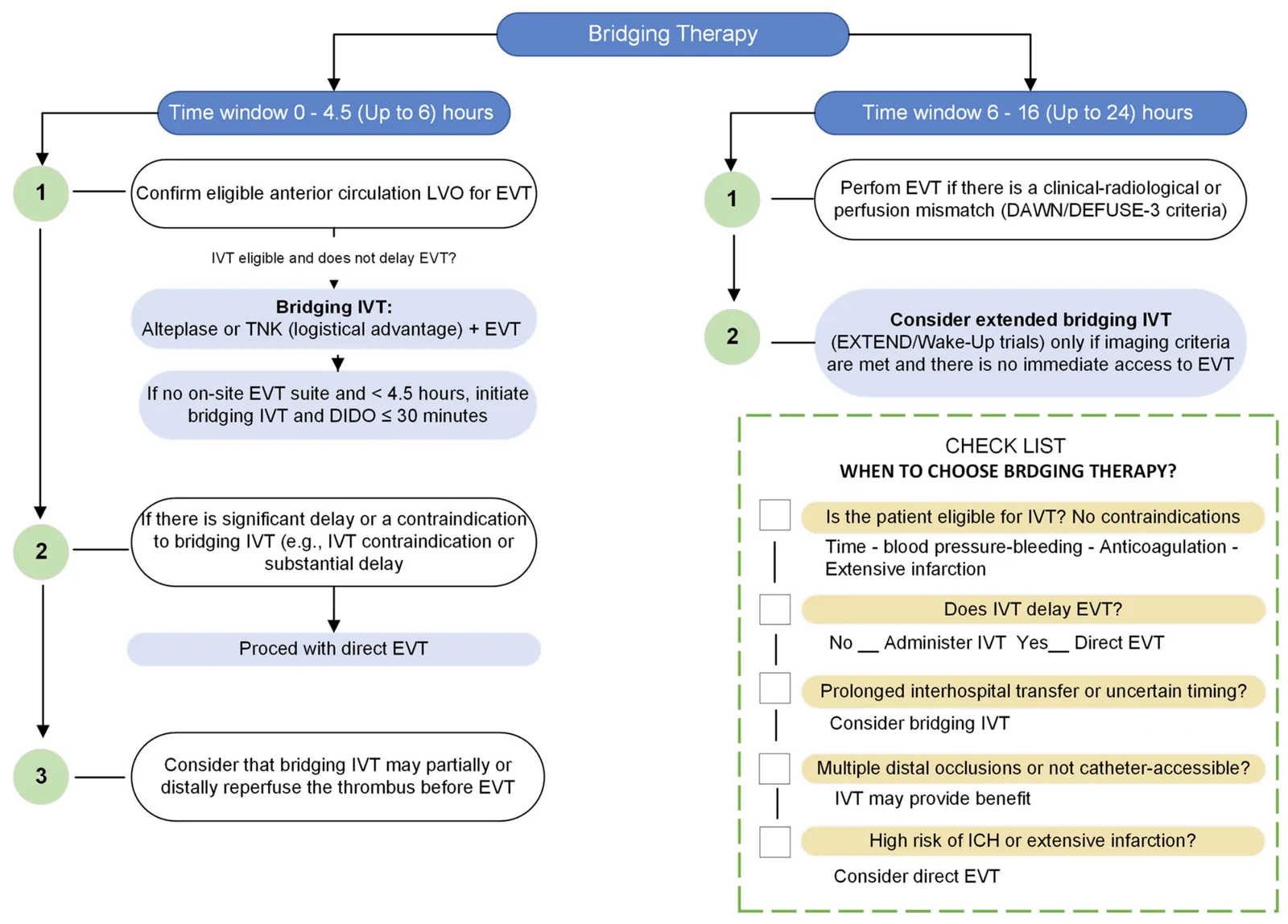
Framework and checklist for bridging therapy decision-making in acute ischemic stroke. Abbreviations: AIS, acute ischemic stroke; EVT, endovascular therapy; IVT, intravenous thrombolysis; LVO, large vessel occlusion; TNK, tenecteplase; DIDO, door-in-door-out; ICH, intracranial hemorrhage.

Although ongoing studies may further refine these strategies, current practice favors a pragmatic approach in which thrombolysis is used whenever feasible and does not compromise timely mechanical reperfusion [41]. Figure 3 presents a conceptual framework and checklist to support decision-making in bridging therapy selection.

### 3.5. Management of Posterior Fossa Stroke (Basilar Artery Occlusion)

Posterior circulation ischemic strokes, particularly those caused by acute basilar artery occlusion (BAO), represent one of the most severe neurological emergencies. Although they account for only 1–2% of ischemic strokes, their natural history without treatment is associated with very high mortality, reported in some series to reach 80–90%, and a substantial risk of severe disability, coma, or locked-in syndrome among survivors [64].

Timely recanalization of the basilar artery is critical for survival; however, diagnosis is frequently delayed due to the heterogeneity and often subtle nature of early symptoms, including vertigo, diplopia, dysarthria, ataxia, and altered consciousness. This variability requires a high index of clinical suspicion, even in patients with low NIHSS scores [65]. For initial evaluation, non-contrast CT combined with CT angiography (CTA) remains the most accessible approach in many settings. When available, MR angiography or comprehensive CTA including the vertebrobasilar circulation is recommended to improve diagnostic accuracy, as standard imaging protocols may prioritize anterior circulation and fail to adequately assess posterior vessels. Importantly, the NIHSS may underestimate stroke severity in posterior circulation events, as symptoms such as isolated vertigo or ataxia can result in low scores despite the presence of potentially life-threatening pathology. Therefore, clinical assessment must be integrated with vascular imaging findings when considering reperfusion strategies [65]. In addition, atypical presentations, including seizures during stroke-code activation, may further complicate early recognition and delay diagnosis, particularly in resource-constrained environments [64,65].

#### 3.5.1. Endovascular Therapy in Posterior Fossa Stroke: Current Evidence

Reperfusion strategies in posterior circulation stroke are generally based on principles similar to those applied in anterior circulation, including intravenous thrombolysis within 4.5 h and consideration of mechanical thrombectomy in cases of basilar artery occlusion or other major posterior circulation vessel occlusions (e.g., P1 segment of the posterior cerebral artery or proximal cerebellar arteries), when appropriate clinical and imaging criteria are met [64]. However, the evidence base in posterior circulation has been historically less robust and more heterogeneous, requiring cautious interpretation.

For several years, randomized evidence for EVT in basilar artery occlusion (BAO) remained inconclusive. Early trials such as BASICS and BEST did not demonstrate a clear benefit, although their interpretation is limited by methodological challenges, including crossover and heterogeneous patient selection. These limitations prompted further studies to better define the role of EVT in posterior circulation stroke:

- BASICS (NEJM 2021): This trial included 300 patients, most treated within 6 h of symptom onset. Functional independence (mRS 0–3 at 90 days) was achieved in 44% of patients undergoing thrombectomy compared with 38% receiving medical therapy (RR 1.18; 95% CI 0.92–1.50; *p* = 0.17). The lack of a statistically significant benefit may be partly explained by trial design factors, including the inclusion of patients with lower stroke severity (NIHSS <10) and a relatively high rate of spontaneous recanalization, which may have attenuated the observed treatment effect [66].
- BEST (Lancet 2020): This Chinese randomized trial was stopped early after enrolling 131 patients (target 344) due to recruitment challenges and a high rate of treatment crossover. Favorable outcomes (mRS 0–3) were observed in 42% of patients undergoing thrombectomy compared with 32% receiving medical therapy (adjusted RR 1.74; 95% CI 0.81–3.74), although this difference did not reach statistical significance [67].

Consequently, for a period of time, thrombectomy in basilar artery occlusion (BAO) was considered a Class IIb recommendation, reflecting uncertain benefit [50]. Despite the neutral results of early trials, both BASICS and BEST suggested a potential trend toward benefit, particularly in patients with more severe deficits (e.g., NIHSS ≥10), which informed the design of subsequent studies. More recently, two randomized controlled trials conducted in China and published in NEJM in 2022 (ATTENTION and BAOCHE) have provided more consistent evidence, contributing to a shift in the overall balance of data:

- ATTENTION (NEJM 2022): This trial included 340 patients with acute basilar artery occlusion (BAO) and NIHSS ≥10 treated within ≤12 h from symptom onset (median 5 h). Functional independence (mRS 0–3) was achieved in 46% of patients undergoing EVT compared with 23% receiving medical therapy (RR 2.06; *p* < 0.001), leading to early termination of the trial for efficacy. Mortality was also significantly reduced (37% in the EVT group vs. 55% in the medical therapy group; RR 0.66; 95% CI 0.52–0.82), with low rates of symptomatic intracranial hemorrhage (5% vs. 0%) [68].
- BAOCHE (NEJM 2022): This trial extended the treatment window to 24 h (median 11 h) and included 217 patients with acute BAO, NIHSS ≥10, and no evidence of extensive infarction. Functional independence (mRS 0–3) was achieved in 46% of patients undergoing EVT compared with 24% receiving medical therapy (*p* < 0.001). Mortality was lower in the EVT group (31% vs. 42%), although this difference did not reach statistical significance, likely due to sample size limitations. Importantly, patient selection excluded cases with chronic occlusion or complete brainstem infarction [69].

Both trials selected patients with favorable imaging profiles, including high pc-ASPECTS (≥8) and absence of extensive bilateral brainstem or cerebellar infarction, supporting the effectiveness and safety of EVT up to 24 h in carefully selected patients [68,69]. Notably, the use of intravenous thrombolysis was relatively low (approximately 25%), and up to 50% of patients required rescue angioplasty or stenting due to underlying intracranial atherosclerotic disease. These findings likely reflect population-specific and pathophysiological differences compared with Western cohorts and should be considered when extrapolating results to other settings.

From a clinical perspective, these findings must be interpreted alongside a key real-world challenge: “the high rate of initial misdiagnosis in posterior circulation stroke”. Despite growing evidence supporting the benefit of EVT in selected patients with basilar artery occlusion, early recognition remains a major barrier. In routine emergency settings, symptoms such as vertigo, ataxia, or diplopia are frequently misattributed to peripheral vestibular disorders, leading to delays in diagnosis and loss of therapeutic opportunities.

This gap between evidence and practice underscores the need for standardized clinical pathways that incorporate specific screening strategies for basilar artery occlusion, along with early vascular imaging when feasible, particularly in resource-constrained environments where access to advanced diagnostics may be limited.

With the emergence of more recent data, recommendations have increasingly shifted toward considering intervention in selected patients. The latest European guideline (ESO-ESMINT 2024) suggests mechanical thrombectomy, in addition to best medical therapy, for patients with acute basilar artery occlusion (BAO) within ≤6 h and also within 6–24 h (Class IIa), provided that specific criteria are met, including significant neurological deficit (e.g., NIHSS ≥10), absence of extensive established bilateral infarction, and appropriate imaging-based selection [11,50].

These guidelines also emphasize that consideration of intravenous thrombolysis should not delay access to thrombectomy, particularly in patients presenting within the endovascular window. When feasible, combined therapy may be performed; however, timely mechanical reperfusion remains the priority. Importantly, these recommendations are conditional and based on low-certainty evidence, reflecting ongoing heterogeneity and residual uncertainty in the available data [11].

In patients with milder symptoms (NIHSS <10), the benefit of EVT has not been clearly demonstrated, and management should be individualized. Nevertheless, given the high risk associated with persistent basilar artery occlusion, some expert opinions support intervention in carefully selected borderline cases when imaging does not demonstrate extensive irreversible injury [50].

#### 3.5.2. Role of IV Thrombolysis in Posterior Circulation Stroke

In basilar artery occlusion (BAO) and other posterior circulation strokes, intravenous thrombolysis (IVT) remains an important component of reperfusion therapy. In many centers, it is administered as the initial intervention even when BAO is suspected, as it may achieve partial or complete recanalization prior to thrombectomy [70]. Early IVT has been associated with improved survival and, in settings without access to EVT, may represent the only available opportunity for reperfusion.

Observational studies suggest that a subset of patients with BAO treated with alteplase beyond 4.5 h may achieve recanalization and favorable outcomes, raising the possibility of a broader therapeutic window in posterior circulation stroke. This may be related to differences in collateral circulation within the posterior fossa; however, these findings are derived from non-randomized data and should be interpreted with caution [71,72].

The ESO-ESMINT 2024 guideline introduced an important update by suggesting intravenous thrombolysis (IVT) for basilar artery occlusion (BAO) up to 24 h from last known well, in the absence of contraindications, particularly when immediate access to EVT is not available. However, this recommendation is based on expert consensus and supported by low-certainty evidence [11]. The underlying rationale is that, in a condition with high mortality in the absence of timely reperfusion, alteplase may offer a net clinical benefit when imaging demonstrates tissue viability. Accordingly, late IVT decisions should be guided by imaging, favoring patients with limited infarction or evidence of viable brainstem tissue, and avoiding those with extensive established injury due to increased hemorrhagic risk [11]. More recently, the EXPECTS trial (NEJM 2025) has provided additional data in this setting. In 234 patients with posterior circulation stroke treated between 4.5 and 24 h after symptom onset (excluding EVT candidates and those with extensive infarction on CT), alteplase was associated with higher rates of functional independence at 90 days compared with standard care (mRS 0–2: 89.6% vs. 72.6%; adjusted RR 1.16; 95% CI 1.03–1.30; *p* = 0.01; NNT = 6), with low rates of symptomatic intracranial hemorrhage and no significant difference in mortality [73]. While these findings support the potential role of imaging-guided thrombolysis in extended windows, they should be interpreted cautiously given the selected population and evolving evidence base.

In regions such as Latin America, where access to endovascular therapy remains limited, extended-window IVT may represent an important therapeutic option in selected patients, ideally guided by multidisciplinary decision-making and imaging confirmation of tissue viability [70,71,72,73].

In summary, the management of posterior circulation stroke requires an integrated and context-adapted approach that includes: (1) early recognition despite atypical or fluctuating symptoms, with a low threshold for vascular imaging in patients presenting with vestibular symptoms or unexplained alterations in consciousness; (2) close monitoring in a stroke unit or intensive care setting due to the risk of rapid neurological deterioration; (3) prompt IV thrombolysis within 4.5 h when indicated; (4) urgent EVT in confirmed BAO in appropriately selected patients, including extended windows up to 24 h based on recent trial criteria; and (5) consideration of imaging-guided IVT in extended windows for patients who are not candidates for EVT. These strategies should be adapted to local resources and system capabilities.

**Figure 4:**
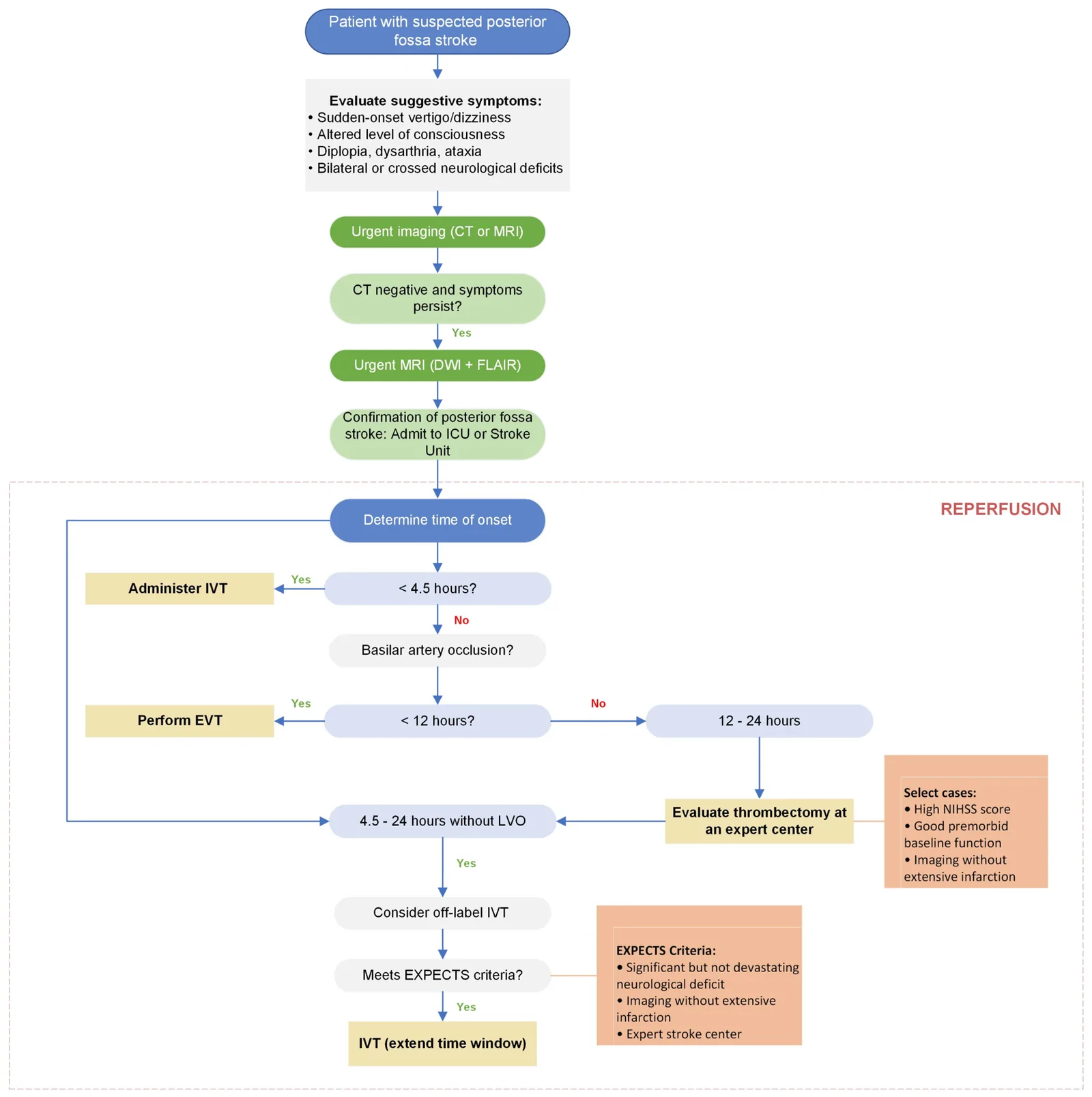
Proposed tiered algorithm for decision-making for suspected posterior fossa stroke, including imaging escalation (CT/MRI), basilar artery occlusion evaluation, thrombectomy time-window assessment, and imaging-guided extended-window intravenous alteplase use in selected non-thrombectomy candidates (EXPECTS-based criteria).

Figure 4 provides a schematic overview of the decision-making process in posterior circulation stroke, integrating IVT and EVT considerations according to time, clinical severity, and institutional resources [14,52].

## 4. Discussion

Recent evidence has substantially transformed the management of acute ischemic stroke, expanding both the population of patients who may benefit from reperfusion therapies and the timeframes in which these interventions can be safely applied. The shift from a strictly time-dependent paradigm (“time is brain”) to a model based on tissue viability (“tissue, and particularly the penumbra, is brain”) has enabled a more individualized approach, allowing treatment of patients who were previously considered ineligible.

Trials such as WAKE-UP, EXTEND, and HOPE have demonstrated that intravenous thrombolysis guided by advanced neuroimaging can provide clinical benefit beyond 4.5 h, and in selected patients, up to 24 h from symptom onset. Importantly, this approach has not been associated with a substantial increase in symptomatic intracranial hemorrhage or mortality in carefully selected populations. This shift toward imaging-based, physiological selection, rather than strictly chronological criteria, has redefined the traditional boundaries of pharmacological reperfusion.

In parallel, mechanical thrombectomy has become established as the standard of care for large-vessel occlusions, initially within 6 h of symptom onset and, following the DAWN and DEFUSE 3 trials, extended to selected patients up to 24 h. More recent studies, including SELECT2 and ANGEL-ASPECT, suggest that patients with large infarct cores may also derive benefit from mechanical reperfusion when selection is guided by objective imaging criteria, such as core volume and penumbral assessment. These findings challenge the traditional view that extensive infarction is uniformly untreatable, highlighting that even partial reductions in disability can have meaningful clinical and societal impact.

The question of bridging therapy versus direct thrombectomy remains an area of ongoing debate. Although overall results suggest comparable efficacy between both strategies, combined therapy is associated with higher rates of early recanalization at the expense of a modest increase in symptomatic hemorrhage. Current guidelines adopt a pragmatic approach, recommending the use of intravenous thrombolysis whenever feasible, provided it does not delay endovascular treatment. In settings with immediate access to thrombectomy, the incremental benefit of thrombolysis may be limited. However, in regions where access to endovascular therapy is constrained, or transfer delays are common, as is often the case in Latin America, bridging therapy continues to play a critical role in ensuring timely reperfusion within comprehensive stroke care systems.

In posterior circulation stroke, an area historically limited by sparse and heterogeneous evidence, recent trials such as ATTENTION and BAOCHE have provided support for the benefit of EVT in selected patients with basilar artery occlusion up to 24 h, particularly in those with NIHSS ≥10 and without extensive established infarction. Complementary evidence from the EXPECTS trial [73] suggests that late intravenous thrombolysis (4.5–24 h) may improve outcomes in selected patients with mild-to-moderate posterior circulation stroke when EVT is not available, and imaging demonstrates salvageable tissue.

Taken together, these findings support a more inclusive, yet still carefully selected, therapeutic approach, potentially expanding access to reperfusion in a clinical setting traditionally associated with high mortality and disability.

Looking ahead, the central challenge lies in translating this evolving body of evidence into effective clinical practice across heterogeneous healthcare settings. In Latin America, technological, geographic, and infrastructure limitations continue to restrict access to advanced imaging and endovascular therapies. Therefore, key priorities include optimizing critical time metrics (door-to-needle, door-to-groin, and door-in-door-out times) and strengthening regional stroke care networks [74,75].

In parallel, the expansion of telemedicine programs, development of stroke units, and investment in interdisciplinary training are essential to bridge the gap between evidence and practice, ensuring that advances in stroke care lead to meaningful improvements in patient outcomes [76,77].

Emerging research is further expanding the horizons of stroke care, including advances in neuroprotection, post-reperfusion hemodynamic management, and the integration of biomarkers and artificial intelligence to predict treatment response and the risk of hemorrhagic transformation. These developments are expected to further refine patient selection and therapeutic strategies, reinforcing a paradigm in which stroke care is no longer defined solely by time, but by the dynamic biological state of cerebral tissue and the need for rapid, coordinated, and personalized intervention.

Overall, advances in extended therapeutic windows, endovascular therapy, and posterior circulation management are reshaping the prognosis of acute ischemic stroke. The remaining challenge is not the lack of evidence, but the effective translation of this knowledge into equitable, efficient, and data-driven healthcare systems capable of delivering optimal care to every patient.

## 5. Conclusions

The management of acute ischemic stroke is increasingly guided by imaging and tissue viability rather than strict time thresholds, allowing broader application of reperfusion strategies. These include extended therapeutic windows, combined approaches such as bridging therapy (IVT + EVT), the use of tenecteplase as a practical alternative to alteplase, and selected use of thrombolysis in posterior circulation stroke.

However, translating these advances into improved outcomes in Latin America remains challenging, as access to advanced imaging and endovascular-capable centers is still uneven. Progress will depend on strengthening referral networks, optimizing key time metrics (door-to-needle, door-to-puncture, and door-in-door-out), and adapting diagnostic and treatment pathways to local contexts.

This review provides an updated synthesis of contemporary reperfusion evidence and proposes pragmatic, context-adapted management frameworks tailored to regional needs. These approaches are intended to support protocol development in stroke units and emergency services across different levels of care, with the goal of improving efficiency, standardizing practice, and reducing inequities in access to reperfusion therapies in Colombia and across Latin America.

## Figures and Tables

**Table 1:** Selected intravenous thrombolysis studies in acute ischemic stroke (standard vs. extended window). Abbreviations: CT, computed tomography; CTP, computed tomography perfusion; MRI, magnetic resonance imaging; DWI, diffusion-weighted imaging; FLAIR, fluid-attenuated inversion recovery; PWI, perfusion-weighted imaging; mRS, modified Rankin Scale; sICH, symptomatic intracranial hemorrhage; OR, odds ratio; RR, relative risk; RCTs, randomized controlled trials. Symbols: ↑, increased risk or frequency; no ↑, no increased risk or frequency.

Study	Time Window	Imaging-Based Selection	Key Result
NINDS (1995)	<3 h	Non-contrast CT (exclude hemorrhage)	mRS 0–1: 39% vs. 26% (OR 1.7); sICH 6.4% vs. 0.6%
ECASS III (2008)	3–4.5 h	CT without extensive infarction	mRS 0–1: 52.4% vs. 45.2% (OR 1.34; *p* = 0.04); sICH 2.4% vs. 0.2%
WAKE-UP (2018)	Unknown onset (wake-up)	MRI DWI-FLAIR mismatch (time-surrogate marker of <4.5 h	mRS 0–1: 53% vs. 42% (OR 1.61; *p* = 0.003); sICH 2.0% vs. 0.4%; no ↑ mortality
EXTEND (2019)	4.5–9 h/wake-up	CTP or MRI PWI/DWI (penumbra >10 mL, core <70 mL, ratio ≥1.2)	mRS 0–2: 35.4% vs. 29.5% (RR 1.44; *p* = 0.04); sICH 6.2% vs. 0.9%; no ↑ mortality
HOPE (2025)	Up to 24 h (≈85–90% treated before 9 h)	CT perfusion–core mismatch (same criteria as EXTEND)	mRS 0–1 at 90 days: 49.5% vs. 35.5%(absolute diff 13.9%; *p* = 0.004); sICH 3.8%; no ↑ mortality
Meta-analysis (2025)	4.5–24 h (8 RCTs, ≈1742 patients; HOPE not included)	Advanced imaging (CTP, MRI PWI/DWI, DWI–FLAIR mismatch)	mRS 0–1: 44% vs. 36% (OR 1.43; *p* < 0.001); mRS 0–2: similar benefit (OR ≈ 1.36; *p* = 0.002)

## Data Availability

The original contributions presented in the study are included in the article; further inquiries can be directed to the corresponding author.

## Abbreviations

ASPECTS	Alberta Stroke Program Early CT Score
BAO	Basilar Artery Occlusion
BP	Blood Pressure
COPE	Committee on Publication Ethics
CT	Computed Tomography
CTA	CT Angiography
CTP	CT Perfusion
CTP/CTA mismatch	Perfusion–angiography mismatch (marker of viable ischemic penumbra)
DIDO	Door-In Door-Out time
DM	Diabetes Mellitus
DNT	Door-to-Needle time
DPN	Door-to-Puncture time
EMS	Emergency Medical Services
EVT	Endovascular Therapy (mechanical thrombectomy)
AF	Atrial Fibrillation
FLAIR	Fluid-Attenuated Inversion Recovery
HTN	Hypertension
SU	Stroke Unit
ICMJE	International Committee of Medical Journal Editors
IVT/rtPA	Intravenous Thrombolysis/Recombinant Tissue Plasminogen Activator
LMICs	Low- and Middle-Income Countries
LVO	Large Vessel Occlusion
mRS	Modified Rankin Scale
MRI	Magnetic Resonance Imaging
NIHSS	National Institutes of Health Stroke Scale
SVO	Video Monitoring System (if applicable in stroke units or monitoring protocols)
TICI	Thrombolysis in Cerebral Infarction (angiographic reperfusion grade)
TIA	Transient Ischemic Attack
TNK	Tenecteplase
